# Between Patriots and Refugees: Negotiating Displacement With the State in (Post-)Second World War China

**DOI:** 10.1177/00220094251396895

**Published:** 2025-12-01

**Authors:** Jiayi Tao

**Affiliations:** 191590 University of Vienna, Austria

**Keywords:** Displacement, nationalism and patriotism, postwar China, refugee history, Second World War

## Abstract

This article examines how non-elite Chinese navigated displacement in relation to the state during the decades of war and revolution in the 1930s and 1940s. The conflicts, including the Sino–Japanese War and the Chinese Civil War, created displacement and complicated identities. This article reveals the diversity of wartime identities, beyond the well-studied identification with the nation and native place. It highlights the reinvention of ‘righteous people’ as a survival tactic to exploit the discourse of nationalism and the complexity of ‘refugees’ not just as a social category or status, but one of the identities people with little social capital strategically rejected or exploited to navigate displacement. In these and other ways, this study complicates the image of non-elite displaced Chinese. They were more than passive recipients of humanitarian aid or resilient survivors that cared little about politics, but were skilful in engaging with political identities for functional purposes.

In 1939, one year before the Chinese Nationalist government established Chongqing as its wartime capital during the war with Japan (1937–45), the city had seen an influx of hundreds and thousands of displaced people. Amid chaos, an association consisting of people from Jiangsu province wrote to the government, urging the elimination of the Chinese term *nanmin* (難民, refugees) from official usage; instead, the term *yimin* (義民, righteous people) should be used as an alternative to make a distinction from those who ‘suffer from natural or human disasters, stranded and waiting for relief’.^1^ The key message delivered here was a self-interpretation of displacement: they did not desperately escape from perceived or actual threats of conflicts, but their relocation to Chongqing was a product of a voluntary, patriotic choice to refuse to live under Japanese rule. After the publication of this request in newspapers, a commentary titled ‘*Nanmin* is not a shameful label’ reminded its readers that Jiangsu natives sought to change the unwelcoming public attitude towards refugees, who were often viewed as a black hole of resources.^2^ The narrative constructed by displaced Chinese is thus best understood as an attempt to exploit the discourse of nationalism to their own advantage, rather than evidence of the breadth and depth of national sentiments. In making this narrative, they rejected the label of victims for a pragmatic purpose of removing discrimination against them.

The centrality of patriotism to this self-interpretative account of displacement was insisted in a context where Chinese nationalism had been a transformative force since the early twentieth century, rationalising war mobilization efforts and an expansion of the state's role. Historians like Stephen R. MacKinnon have examined how the refugee crisis during the Sino-Japanese War led to humanitarian activism and prompted the government to become more involved in relief affairs, for example, sponsoring land reclamation projects.^3^ This body of research often adopts a top-down approach, focusing on politicians and social elites alike as aid providers. As a notable exception, R. Keith Schoppa explores refugees’ experiences in the unoccupied areas of Zhejiang province, emphasizing the lack of ‘overt nationalism or patriotic fervour’ among them and arguing that ‘the nation might just not be in the picture’.^4^ This article seeks to continue this refugee-focused scholarly effort, which a burgeoning scholarship on refugee history has urged.^5^ It takes a further step to consider how the complexity of war created different patterns of interaction between displacement and nationalism, which, in this article, refers to a discourse on national survival and reconstruction. This discourse was more than rhetorical; it critically underpinned the expansion of government aid initiatives. State power in the southwest of China where the wartime capital was located, was less fragile than it was in unoccupied Zhejiang. Also, millions of Chinese men and women benefited from state-led relief efforts, especially after the Nationalist government received a substantial amount of aid from the United Nations Relief and Rehabilitation Administration (UNRRA), created in 1943 with the hope of remaking the war-torn world with the spirit of international cooperation and respect for national sovereignty.^6^ By the time UNRRA terminated its operations at the end of 1947, its aid had assisted over one million internally displaced Chinese through a Nationalist state agency, the Chinese Relief and Rehabilitation Administration (CNRRA).^7^ This article examines how non-elite Chinese negotiated displacement in relation to the state during the Second World War and its immediate aftermath.

Established on a recent scholarly effort to look beyond the analytical framework of the Second World War that was marked by a definite end in 1945, this article emphasizes displacement as a key, often recurring, experience of the Chinese population during the fraught decades of the 1930s and 1940s.^8^ The first wave of mass displacement began with Japan's invasion of Manchuria in 1931. Six years later, the outbreak of the Sino–Japanese conflict as a total war forced the Nationalist government to evacuate west, accompanied by the flight of millions of civilians from a spectrum of social classes. Following the onset of the Pacific War in December 1941, Nationalist-controlled China witnessed the inflow of overseas Chinese fleeing the Japanese advance in Southeast Asia. The Nationalist government failed to resume full control over China after Japan's surrender. The subsequently resumed civil war (1927–37, 1946–49) with the Chinese Communist Party (CCP) continued to create displacement both within and from China. Before Asia saw more waves of mass displacement during the Cold War, the withdrawal of the Nationalists to Taiwan in 1949 had populated the island with large waves of displaced Chinese.^9^ The wide variety of displacements in these interconnected episodes complicated the identities people actively engaged with, which included more than the national identity and identification with their native place. I argue that refugees were more than a social and bureaucratic category or a status, but one of the war-induced identities people rejected or exploited at different occasions to navigate displacement. In doing so, this article brings new layers of complexity to the term ‘refugee’, which historians of China have so far often used in a narrow sense as ‘victims of wartime violences’.^10^

The worlds of elites have shaped much of our understanding of China's Second World War ‘beyond the battlefield’.^11^ While elites’ writings provide valuable insights into how individuals made ‘a greater sense of order’ to the experience of displacement,^12^ this article looks beyond their worlds and uses institutional records and newspaper sources to focus on non-elite displaced Chinese. Unlike diaries and memoirs, letters addressed to government agencies and the press often served functional purposes such as requesting aid. To be sure, the ability to write was a privilege at a time of high illiteracy in China, but in these cases, the authors extended their activities beyond intellectual circles and represented those who had limited social capital to draw upon, after their lives had been uprooted.

The petitions and complaints that will be analysed in this article present a different image of non-elite displaced Chinese from the ones in contemporary writings by elites and historical research. Having worked for UNRRA in war-devastated Hubei and Hunan provinces in 1946, American George Hodel remarked in his memoir that ‘the sight of dead bodies in the streets became so common that no one turned to look’, and in the faces of the starving population, ‘one saw death – perhaps today, perhaps tomorrow’.^13^ Albeit serving a unique purpose of glorifying UNRRA as a lifesaver that brought about hope, these words epitomized the prevailing writings in which the image of Chinese refugees was single and unified. The refugee was an incarnation of suffering and victimhood, through which to call for humanitarian aid or condemn the brutality of war and the incompetence of the Nationalist government.^14^ Historical research has emphasized displaced people's resilience, notably the capacity to resume life after displacement.^15^ This article further complicates the image of non-elite displaced Chinese, who were, in many cases, skilful in making their desires heard. They were far from passive and speechless beneficiaries of worldwide diplomatic, internationalist, nationalist and bureaucratic efforts at managing global displacement.

Although individual journeys were not unusual and family remained a core social form, this article narrows its focus to the cases where people acted collectively and in ways that transcended family networks. It consists of two sections. The first section examines the evolution of the righteous people and refugee identities, showing that the nation was often in the picture. The second section focuses on a cohort of overseas Chinese mechanics who had joined China's war effort. Some were later descended to refugee status. This case is unusual, because they possessed a rare ability to draw on politicians as their proxy agency.^16^ It, however, carries a wider significance in revealing how war-induced identities were strengthened through collective action and government measures (and their absence thereof). This article is, after all, about how displaced people struggled for a place in a war-torn world where nations were in the making.

Throughout the last few centuries, the Chinese term *nanmin* carried a consistent meaning as victims of natural or human disasters, while the meaning of *yimin* was in flux. Invoking righteousness (*yi*, 義) from the pool of Confucian values, Ming and Qing governments routinely conferred *yimin* as a title on civilians who helped preserve order during times of crises, such as self-organized militia against rebels.^17^ This government practice to foster loyalty gave the term *yimin* a degree of prestige, which was nevertheless undermined by the anti-foreign and anti-Christian Boxer Uprising at the turn of the twentieth century. Righteousness was key to the self-representation of the rebels, and in some cases, they concisely referred themselves to *yimin*.^18^ In response, Confucian elites condemned these people – what they called ‘bandits’ – for misusing the concept of righteousness, which should serve to preserve social hierarchy and loyalty to the Qing government and the nation.^19^ In this sense, *yimin* functioned as an arena for model citizenship. Competing interpretations notwithstanding, it embodied an invitation for individuals to think of themselves as components of a larger community and voluntarily take part in shaping the locality, if not the nation's future, in a constructive way.

Although some still held the value of Confucianism, it was increasingly discarded by Chinese intellectuals, some of whom would later decide China's refugee policy. In 1905, the Confucian-based imperial examination system was abolished after operating for over a millennium to select officials. Jiang Tingfu, later Director of CNRRA, was one among many who subsequently followed a Western curriculum and went abroad for further study.^20^ The New Culture Movement of the 1910s was filled with radical calls to eliminate Confucianism. This, however, was followed by a conservative pushback aimed at restoring traditional values. When Nationalist politicians launched the New Life Movement in 1934, righteousness resumed its usefulness to rulers: it now helped produce patriotic and healthy Chinese citizens.^21^ It is noteworthy that the meaning of *yi* did not narrow down to patriotism. Along with other intertwined Confucian virtues (propriety, integrity and shame at wrongdoing), it served to promote citizenship and achieve the ultimate goal of ‘national reconstruction’, or at least saving the nation from Japanese aggression.^22^ Officials sometimes invoked a Mencius saying *she sheng qu yi* (捨生取義, give up one's life for the value of righteousness) to encourage the choice to place national interests ahead of personal ones when these two are in conflict.^23^

If New Life advocators associated sacrifice for the nation with the old merit of righteousness, *yimin*, as a set phrase, remained outside the New Life lexicon. It yet referred to anti-Japanese resistance activity. By then, Japan's control of Manchuria had caused waves of displacement to the south, after which humanitarian activism spread across China's major cities. Whereas fundraisers preferred the term *nanmin* to highlight the victimhood of forced Chinese migrants to evoke sympathy, *yimin* exclusively alluded to the anti-Japanese ‘righteous armies’ (*yiyongjun*, 義勇軍) active in Manchuria after 1931.^24^ Rana Mitter has pointed out that this name, ‘strongly implying volunteers rather than press-ganged recruits’, sought to encompass various resistance forces, including bandits, former warlord soldiers and students.^25^ The distinction between *nanmin* and *yimin* was kept by those who insisted on their own identity as the latter – as fighters rather than victims – and those who saw the usefulness of the image of volunteer corps for promoting the cause of national salvation.

The drastic turn of the Sino–Japanese conflict into a nationwide total war in 1937 expanded the meaning of ‘righteous people’. In May 1938, when millions of urban dwellers and peasants were fleeing from the Japanese occupation of areas in the east, Xu Shiying, a top-level relief official of the Nationalist government, argued in a newspaper article that ‘refugees are indeed righteous people’. From the perspective of policymakers, he made this point to urge his colleagues to give refugees not only material relief but also ‘emotional support’, in ways such as framing their displacement as an ‘honourable’ choice to prefer starvation to living under occupation.^26^ Nationalism was proposed as a psychological remedy for trauma. Xu's point was echoed by a few propaganda pieces, but opposing opinions existed. One argued that refugees would only be acknowledged as righteous people after having proved their willingness and capacity to work; otherwise, they were just ‘lazy people’ who should be condemned.^27^ This point of view reveals a mixture of expectation and contempt imposed on displaced Chinese who were likely to descend to refugee status. Compared to Japanese repatriates who faced discrimination because of their exposure to ‘unhygienic conditions’ in former colonies, ‘refugees’ in China were similarly a source of anxiety over public health.^28^ In the late 1930s, when China's national survival was at stake, the negative connotation of ‘refugees’ further involved a critique for not assuming a more active role in the war effort. Amid this controversy, displaced Chinese across a spectrum of social classes sought to keep a distance from the category ‘refugees’. A range of old and new collective identities prevailed. The long-existing label ‘people from downriver’ (*xiajiangmin*, 下江民) now highlighted a shared experience of relocation from the lower and middle Yangzi River without indicating a downward social mobility or even any connection to the war.^29^ Some other identities did not avoid the context of war. Whereas *nanbao* (難胞, refugee compatriots) constructed refugeedom as a collective, national experience, *yimin* was more specifically targeted at reshaping displacement into an act of patriotism that contributed to China's war effort. Jiangsu natives, as has been shown in the beginning of this article, took a further step to ask the Nationalist government to replace *nanmin* with *yimin* in official vocabulary.

While offering a sort of sense and social position in a sea of uncertainty, many of these war-induced identities were constructed through the imagination of Others. Displaced Chinese defended their righteousness by juxtaposing with traitors and ‘docile people’ (*shunmin*, 順民), those who lived under Japanese occupation.^30^ As Chinese historians have shown, the government's condemnation of *shunmin* became stronger in a context where many refugees chose to return to their hometowns in Japanese-occupied areas.^31^ Just as refugees’ flight from the Japanese was entangled in a nationalist discourse, the return movement was considered a mild form of betrayal to the nation. Later, the civil war context would continue giving political meanings to refugee flows into and out of Communist-controlled areas. To take another example, downriver people were imagined in contrast to upriver people, that is, natives of Chongqing or the Sichuan province, where Chongqing was located. As the war produced diverse identities and social divides, some nationalists called for the cultivation of national solidarity. A newspaper article argued that people under Japanese occupation were likely to be poor patriots who could not flee home, and it was thus a lack of vision to award only displaced Chinese as righteous people.^32^

In response, the Nationalist government took a moderate approach. Rather than forming a policy, it issued an order allowing relief organizations to choose either *yimin* or *nanmin* as a referral to aid recipients.^33^ In practice, the two terms were used almost interchangeably.^34^ Relief workers, many of whom had also been displaced, helped turn *yimin* into a label for the destitute displaced population in Nationalist-controlled China by naming shelters and soup kitchens as ones opened specifically for *yimin*. In the meantime, self-help organizations that used *yimin* as a defining keyword began to emerge among displaced Chinese, seeking to ‘promote mutual interests and prompt the government to act’ in their interests.^35^ These practices linked this label closely to lower-class displaced Chinese, undermining its potential to evoke national feelings across wartime borders and social classes.

The ambiguous use of *yimin* and *nanmin* persisted into the immediate aftermath of Japan's surrender, when the state agency CNRRA was created as the distributing agency of the UNRRA China aid, the largest amount of aid among UNRRA's beneficiary countries. Across China, CNRRA officials used either term to translate what was called ‘internally displaced people’ in UNRRA's vocabulary of relief. In early 1946, CNRRA's headquarters requested its regional offices to abandon the term *yimin* and adopt *nanmin* on all occasions, because the former was an ‘ordinary’, or unprofessional, term.^36^ Unlike the New Life Movement promoted by an anti-communist cadre of Kuomintang politicians, the international dimension of China's relief and rehabilitation undertakings led to the rise of American-educated elites into CNRRA's leadership. They had little interest in reinventing Confucian values. We also need to note that the significance of UNRRA – and the spirit of internationalism it encouraged – was established through recognising and offering solutions to postwar ‘problems’ that plagued both Europe and Asia.^37^ One goal was to bring an end to millions of people refugee status, rather than righteousness as a merit. This terminology policy was underpinned by the issuance of ‘refugee certificates’, recording particulars including the name, gender, age, profession and native place (Figure 1). The other side of the certificate states that relief officials must write down all sorts of official aid (i.e. free transportation, accommodation, relief supplies and cash aid) received by the refugee. This gives us a clue about the practical benefits associated with the refugee identity.

**Figure 1.**
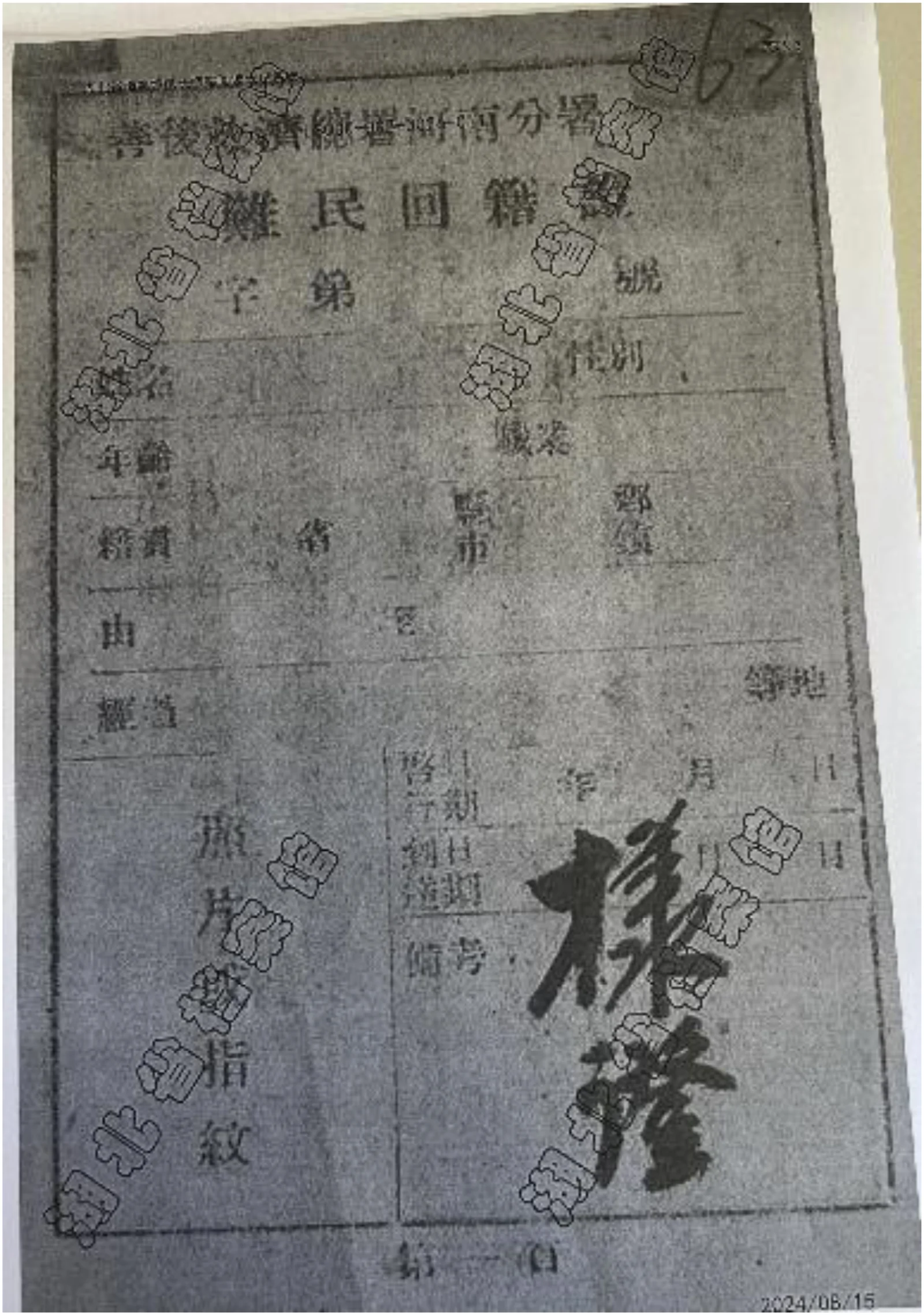
A sample refugee certificate issued by the CNRRA Henan office, undated, LS030-002-0026-011, Hubei provincial archives.

Within the expanding sphere of official aid, displaced Chinese had varying attitudes towards the refugee identity. Not all of them saw it as a stigma and rejected it. Some comfortably lived as refugees, or, as CNRRA officials complained, turned refugee status into a livelihood, moving between different refugee shelters once they had reached the maximum allowed duration of stay.^38^ While the certificate system was designed to avoid the duplication of relief efforts, in reality, refugee certificates were traded.^39^ People exploited the incapacity of the Nationalist bureaucracy to run a truly identity-document-based national system.

As these survival tactics reinforced the stereotype of refugees as lazy and ‘useless’, righteous people continued to exist as an identity that lower-class displaced Chinese insisted on and exploited. This should be understood in a context where CNRRA's regulations did not apply to the Nationalist bureaucracy as a whole. Local governments continued calling internally displaced Chinese *yimin*. In a final report published by the municipal-level Wuhan Temporary Relief Committee in July 1946, *yimin* was adopted to refer to those who received food, cash, medical or housing aid in this transit site on their way home.^40^ At the level of the central government, the Ministry of Social Affairs, at the same administrative rank as CNRRA, circulated regulations in December 1946 on services to help *yimin* return home.^41^ This inconsistency within the Nationalist government was a testament to the inertia of wartime relief practices. Even the CCP propaganda contributed to the persistence of the *yimin* identity. In 1946, a newspaper report published in the CCP mouthpiece *Xinhua ribao* began with a sigh that in Nationalist-controlled areas, ‘*yimin* are descending into *nanmin*’; unsurprisingly, this article does not emphasize patriotism as a motive for migration, only portraying how the Nationalist military expelled, dispossessed and conscripted the displaced population.^42^ It shapes a contrast with what we shall next see: the voice of the displaced Chinese themselves.

In May 1946, the Shanghai-based newspaper *Shenbao* published a well-crafted and moving letter written on behalf of over 1,000 homeless displaced people residing in a government-run camp. Titled ‘Righteous people's appeal’, it did not include any individual story but began with a narrative linking their current plight with the earlier decision as ‘ordinary patriots’ to follow the government's evacuation at the expense of their ‘employment and family ties’.^43^ It went on to state that they were so ‘ordinary’ like *nisha* (泥沙, mud and sand, a metaphor for historically insignificant individuals) and could do no more for national salvation than to protect the value of righteousness through the life-risking act of fleeing from Japanese rule. This letter contrasted their past behaviour as model citizens not just with ‘docile and cowardly people’ under Japanese occupation but also with their current ‘uselessness’ to the state, because they ‘do not have expertise for the project of “national reconstruction” or knowledge to help make a “democratic’ state’.^44^ In this way, the appeal letter showed a deep understanding of a shift in the Nationalist government's relief policy: displacement caused by the Japanese lost its political importance, especially at a time when it hoped to quickly rebuild relations with Japan in the face of the Chinese Communist threat.^45^ In practice, CNRRA officials extensively employed the displaced and destitute population as labourers in short-term rehabilitation projects like restoration of highways and railway roadbeds.^46^ If the government saw relief as a reward to model citizens in the era of reconstruction, the letter presented a powerful alternative that perceived relief as a government duty and established the collective experience of displacement as the path to China's victory, on the basis of which the state-building project could survive and continue.

In this case, righteousness was claimed was asserted not in response to public discrimination, but as a justification for making a set of demands to the government: (1) subsistence; (2) establishing literacy classes for children, a sports field, a barber's room, a bathroom and a clinic; and (3) providing jobs or opportunities for collective production.^47^ These multilayered requests reveal that this group of people considered it the government's responsibility not just for feeding and housing them but for helping them become economically self-reliant and socially integrated. Historians like Mitter have shown that the transnational context during the Second World War made Nationalist officials realize the necessity of increasing the government's role in social provision.^48^ Here, I highlight that this increase was also urged from the below. To take the case of Wuhan 1938 as an example, the government aid was framed as a temporary measure, with emphasis placed on the evacuation of refugees to safer zones, as a matter of routine in earlier times of crises.^49^ Yet, the protracted war enabled the transformation of humanitarian norms in Nationalist-controlled China. Relief workers began to pursue ‘longer-term change’, educating displaced men and women to be consciously committed to the goal of national resistance and assigning them to state-owned factories and cooperatives.^50^ The returnees who had been exposed to a combination of relief and war mobilization efforts carried with them heightened expectations of governmental aid and helped spread them to a larger audience.

Anxiety was apparent throughout the letter, because there was a rumour that all government-run camps would soon be closed. Before writing to the press to call for attention, this group had sent a representative to the CNRRA office but received a vague response.^51^ By identifying with ‘righteous people’ in the open letter, they framed their plight as a widespread social issue that the government had the responsibility to address. At that time, urban-based refugee shelters were the only place for many returnees who were unexpectedly caught in limbo. Some could not, or did not want to, return to their hometowns that were now under CCP control. Another scenario frequently occurred: people returned home after years of displacement only to find their houses destroyed and ‘properties, even farming tools’ gone; with no way to start a new life, they returned to shelters and became displaced people again.^52^ Remarkably, anxiety did not turn into a sense of helplessness in this letter but into a forceful tone: the final line warned that if the Nationalist government failed to meet the demands, their very presence would do harm to social stability.^53^ If the previous texts presented an image of politically conscious and obedient patriots, the subtext was imbued with rage and agency: we have survived the war with Japan and thus dare to act collectively against the government to achieve our goal.

Letters of petition poured into the press, government agencies and UNRRA's regional offices. The authors knew how to address different audiences. The United Nations archives record a letter from a group of 200 returnees who had found their pre-war houses destroyed and resided in the Jiangsu guildhall in Hankou, asking for food aid. This letter followed a Nationalist bureaucratic format, with an abstract of the request and reasons at the beginning and personal seals or fingerprints placed at the end (Figure 2). The authors identified themselves more frequently with ‘righteous people’ than with ‘refugees’. This was probably more an attempt to follow a social norm than to employ a strategy, because they did not adopt a patriotic narrative, only presenting their frantic and desperate flight away from the Japanese in 1937, gratitude for the Allied war effort and their current difficulties due to inflation. Having overlooked the subtle difference between *yimin* and *nanmin*, the interpreter hired by UNRRA translated both terms into ‘refugees’.^54^ UNRRA officials urged their CNRRA colleagues to act, but had no intention to investigate their displacement experience; nor do the full names of representatives lead us to more information. Yet, this must be one among many attempts of the displaced group to petition for aid, and during the process, the literate cadre became more skilful in making narratives and presenting requests.

**Figure 2.**
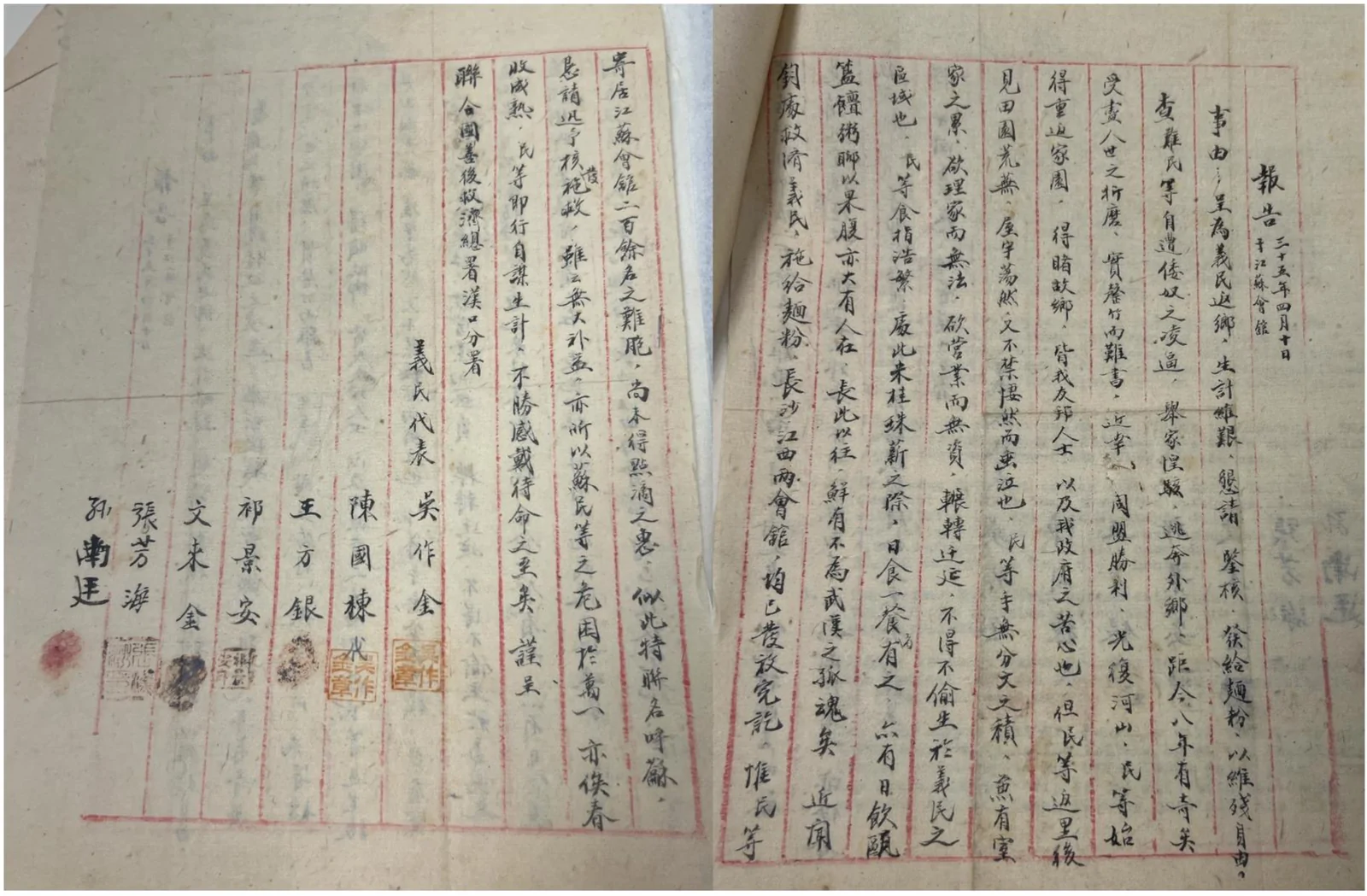
Letter by representatives of a group of displaced Chinese to the UNRRA office in Hankou, 10 April 1946, S-1148-0000-0114, United Nations archives and records management section (New York City, NY).

The mass displacement in China's Second World War led millions of people to experience a drop in social status. Yet this shared experience barely altered the negative connotations associated with refugees as *nanmin*; instead, another term *yimin* was jointly re-invented by relief workers and displaced Chinese as an alternative emphasizing model citizenship. In the 1950s, as Dominic Meng-Hsuan Yang has shown, the Nationalists in Taiwan used ‘righteous compatriots’ to honour evacuees from mainland China.^55^ In this way, righteousness was refashioned to absorb anticommunism as a core value. While Yang has well situated the case of Dachen refugees in Cold War politics, the ways in which displacement was interpreted and navigated had their roots in the previous decade of the total war, and so did the Nationalist strategy to treat refugees as human resources.

The letters that have been analysed are revealing, but they only provide glimpses into moments of petition. I will next focus on a better-recorded case, that of a group of overseas Chinese ‘volunteer’ mechanics whose displacement experiences were deeply involved in China's war effort with Japan and the politics of the ensuing civil war. It offers a more complex and evolving account of how displaced Chinese formed and exercised collective agency in relation to the state, in addition to engaging with the righteous people and refugee identities.

The Nationalist government's evacuation to Chongqing in 1939 was accompanied by its loss of access to the sea. The newly built Burma Road linking Yunnan province and British Burma then became a major route along which Nationalist-controlled China received resources from overseas and exported raw materials for foreign currency. To overcome the critical shortage of drivers and mechanics, the Federation of China Relief Fund of the South Seas (*Nanyang huaqiao chouzhen zuguo nanmin zonghui*, 南洋華僑籌賑祖國難民總會), a Singapore-based fundraising organization sourcing contributions from overseas Chinese communities for China's refugee crisis, recruited and helped train over 3,000 labourers.^56^ In this article, I use the Nanyang mechanics to refer to these people, stressing the fact that they were brought to China by the Federation to take on the role of skilled workers. This aid-giving craze was both a product of the enthusiasm of overseas Chinese elites for investing in China's political future and a humanitarian ‘tradition’ that had spread across almost all social classes and races in the Straits Settlements since the 1870s.^57^ Just as different anti-Japanese resistance forces in Manchuria were reduced to a single image of volunteer corps, ‘volunteers’ labelled the recruited mechanics from the outset, with diasporic patriotism being indicated as the motivation.^58^ By 1945, only two-thirds of the mechanics survived the war. Nearly half of the rest remained in China, and the other half returned to their pre-war residences.

This tragic yet heroic episode has been incorporated into official narratives of China's Second World War in both mainland China and Taiwan, as the governments wished to strengthen their links with overseas Chinese communities through shared memories.^59^ This incorporation was also made possible by the individuals who wished to make their life stories known to more people. In 1985, one of the Nanyang mechanics, Cai Hanliang, visited historians at Huaqiao University in Quanzhou with some personal papers.^60^ His visit inspired scholars in post-Mao China to reach more survivors, conduct oral history interviews and offer new perspectives on the Sino–Japanese War beyond a CCP-centred framework, though patriotism remained a key theme.^61^ Meanwhile, Anglophone historian Ernest Koh uses these mechanics’ transnational life to reveal multiple Second World Wars in Asia, and in doing so, he moves beyond a single patriotic narrative.^62^ Yet, Koh's research has not covered the later stages of their wartime experiences, following the closure of the Burma Road in 1942 due to the Japanese occupation of Burma. The state agency with which the mechanics had been affiliated, the Southwest Transportation Bureau (*Xinan yunshu shu*, 西南運輸署) under the army system, was dissolved.^63^ They thus became unemployed, at a time when Nationalist China saw an influx of millions of displaced overseas Chinese from Burma, Malaya, Hong Kong, the Philippines and other regions that had just come under Japanese occupation.^64^

Remarkably, the Nanyang mechanics remained organized before some could return home in Southeast Asia. Despite speaking different dialects, including Hakka, Cantonese, Southern Min and Hainanese, and coming from British Malaya, the Dutch East Indies, Thailand and the Philippines, this group shared a set of experiences: they were mobilized by the same relief organization, serving the same Nationalist agency and confronting the challenges along the Burma Road, such as malaria and treacherous road conditions.^65^ In addition, a sense of belonging to ‘overseas Chinese mechanics’ was strengthened by government measures like arranging them into a separate unit from those recruited from China. Another example is the creation of a ‘self-help association’ in 1940 under the Nationalist initiative to strengthen a sense of Chineseness among these ‘returnees’ (while some had never been to China before, this group, as overseas Chinese, were regarded as returning to the ancestral land).^66^ The association offered recreational facilities in everyday life and published a weekly journal, which circulated party doctrines, forwarded reports such as the spread of ‘patriotic fervour’ across British Malaya and educated the mechanics to cease unhealthy habits like smoking.^67^ The effectiveness of this publication is doubtful, because some mechanics were illiterate. Still, as historian Xia Yuqing has shown, the association offered a space within which the self-help spirit was encouraged; to take one example, those who could write helped their illiterate colleagues write to their families.^68^ These measures helped shape an exclusive identity that was constituted by a range of social categories: returnees, patriots, overseas Chinese and skilled workers.^69^ Embodying a distinct wartime experience, different from conscripted soldiers or those who fled the war, this collective identity had a strong appeal.

After the closure of the Burma Road, proficiency in English and pleasant personalities secured some mechanics employment, but others were less fortunate. In Kunming, one end of the Burma Road, a cadre of the Nanyang mechanics visited a newly created association, the Overseas Chinese Self-help Association in Yunnan Province (*Yunnansheng huaqiao huzhu lishihui*, 雲南省華僑互助理事會, hereafter the Yunnan Association). They sought assistance for their jobless, homeless, and destitute fellows.^70^ In 1944, the Yunnan Association began to register the Nanyang mechanics who had been scattered across the Burma–India–China theatre; with this re-established network, those employed by the Nationalist army's transportation sector and by US forces in the theatre sent their salaries to those unemployed.^71^ In this way, the mechanics strategically embedded themselves in a larger self-help network of displaced overseas Chinese while preserving their own collective identity. Using the funds from the Nationalist government, the Yunnan Association established a shelter for those who lost sources of income and opened a primary school for children of overseas Chinese.^72^

The Allied victory sparked mass celebrations, which, in turn, created opportunities for people to discuss their postwar futures. At that moment, over 300 Nanyang mechanics who had worked for the US army again became unemployed. In the name of celebrating victory, the Yunnan Association gathered a total of over 500 mechanics stranded in Kunming in October 1945. During this gathering, many expressed their hope for a return to their pre-war residences in Southeast Asia. In addition to family reunification, unemployment was a primary incentive. A consensus was then reached that the mechanics should act collectively to force the Nationalist government to assist their return.^73^ An ensuing petition letter that was endorsed by all was skilfully written. It started with the very reasons why the Nanyang mechanics were now stuck in China: the Burma Road was so difficult that drivers in China dreaded taking it, and as patriots and skilled workers, they responded to the government's call to ‘return’ to China, ‘abandoning their properties, families and comfortable lives’. After highlighting their current willingness to return home, the letter outlined the multiple benefits to the government if it aided their repatriation. It would, firstly, help build the government's prestige; secondly, bolster overseas Chinese communities’ loyalty to China; and lastly, avoid further trouble. The last point was followed by an explanation: it was extremely difficult for them to find jobs in China, owing in part to the lack of support from family and friends and in part to cultural differences; they would inevitably become unemployed and homeless, undermining both social stability in China and the Nationalists’ image among overseas Chinese communities.^74^ Compared to the ‘righteous people's appeal’ in *Shenbao*, the mechanics adopted similar tactics, such as using a patriotic narrative to turn government aid into a responsibility and weaponizing their jobless status as a threat. Moreover, they harnessed their distinct position as a symbol of strengthened ties between China and overseas Chinese communities, whose remittances had long served and would continue serving as a crucial source of income for the Nationalist government. In this way, the mechanics presented their fate as a test for the Nationalist government's capacity and humanity and a crisis that deserved immediate action.

The petition was later sent to Song Ziwen, premier of the Nationalist government, leading to a coordination meeting among bureaucracies, including the Ministry of Foreign Affairs, CNRRA and the Overseas Chinese Affairs Commission, the ministry-level agency responsible for managing overseas Chinese in China. The process was nevertheless long and drawn out because it entailed negotiations with the receiving governments on permission for re-entry. As Meredith Oyen has argued, Southeast Asian governments tended to welcome the return of self-supporting repatriates, rather than those dependent on the UNRRA aid.^75^ In early 1946, having received no updates, the Yunnan Association sent representatives to Chongqing to petition and wrote to the Federation that had brought them to China. He Baoren, a Singapore-based educator who had fled to Chongqing since the outbreak of the Pacific War and a leading figure in the creation of the Federation, urged the Nationalist government to expedite the repatriation of Nanyang mechanics through a proposal submitted to China's National Political Consultative Conference.^76^ Although it is difficult to judge the effectiveness of the pressure exerted within the Nationalist government, we can see the Nanyang mechanics’ effort and capacity to mobilize social leaders and bring their needs to wider attention to combat bureaucratic inefficiency.

This effort had multiple consequences, some of which were not welcomed. In June 1946, the Federation organized a meeting in Singapore to discuss how to press the Nationalist government to send the mechanics back. At this meeting, Tan Kah-kee, chairman of the Federation, contrasted the mechanics’ sacrifice and patriotism with the government's indifference to their present hardship; his speech was circulated through a newspaper article titled ‘All overseas Chinese across British Malaya are outraged at returned mechanics’ homelessness’.^77^ By then, Tan had become increasingly critical of the Nationalist government and sympathized with the CCP. A Yan’an-based newspaper reported the Federation's criticism of the Nationalists but in a harsher, exaggerated tone: ‘the Nanyang mechanics who have contributed to China's war effort are now abused by Chiang Kai-shek's government’.^78^ Being implicated in a civil war context, the mechanics’ response was twofold. While continuing to seek financial aid from the Federation, they refused to be utilized as a political weapon, denying in the press that they had represented political activists in British Malaya.^79^ This announcement, whether its purpose was to appease the Nationalist government or not, exhibits the conflict of interests between the mechanics and the actors they mobilized. The war volunteers now cared much less about China's political future than their own destiny.

The politicization of the Nanyang mechanics’ predicament did expedite government action, as this cohort was now a nuisance. In August 1946, CNRRA expressed the hope to UNRRA to repatriate these people ‘as soon as possible’ because of ‘their miserable living conditions’; and to include them within UNRRA's operational scope as displaced persons, the letter of request underscored that they came to China ‘originally as civilian workers to help with the prosecution of the war against Japan’.^80^ Eventually, with UNRRA's assistance, the first wave of repatriated mechanics arrived in Singapore in November.^81^ If the Nationalist government knew all too well how to make use of international aid for its own benefit, displaced Chinese were also adept at harnessing the government to act for them.

In contrast to the mechanics’ cautiousness in avoiding deep involvement in the civil war, some displaced groups boldly sought to manipulate the Nationalists’ civil-war concerns. When CNRRA was about to terminate its operations at the end of 1947, representatives of self-proclaimed refugees in a CNRRA-run camp wrote a petition for cash aid to the Shanghai City Council, skilfully interpreting their displacement and homelessness as an unfortunate result of their rejection not only of Japanese but also of Communist rule.^82^ In doing so, they simultaneously identified with refugees to emphasize their victimhood and claimed righteousness to urge the government to reward their loyalty, now not to China, but to the party. At that time, the phenomenon of mass exodus from CCP-held areas added a new layer of political significance to ‘refugees’ as a category, thereby expanding people's arsenal in negotiating with the government.^83^ Their struggle helped preserve the notion of righteousness as politically relevant into the Cold War years.

The outcomes of lower-class displaced groups’ protests and petitions varied, often depending on geopolitical factors. If the Nanyang mechanics who, fortunately, secured aid to repatriation lay at one end of the spectrum, at the other end was the situation where government officials ignored the requests from below or refused to act in a concrete way, usually in areas firmly under Nationalist control.^84^ Across the spectrum, the displaced groups’ action was responded to. It sometimes radicalized the government's relief measures. For example, in Wuhan, at whose outskirts gathered a well-equipped CCP army, a group of several hundred displaced Chinese ‘camped overnight’ in the CNRRA office and claimed to stay until their request for flour was met. In a strained atmosphere, they ‘beat up’ the deputy head of CNRRA's regional welfare office. Following this incident, CNRRA began a general registration of all refugees to send off non-Wuhan natives.^85^ Such violent scenarios not merely gave weight to the ‘threats’ frequently expressed in refugees’ writings but were integral to their attempts to negotiate displacement, and more broadly, the conception of government provision at a time of war and revolution.

People with different social backgrounds had varying capacities to navigate displacement, which was often accompanied by dispossession. Equipping with technical, artistic and multilingual skills usually meant a greater opportunity for employment and resettlement (or repatriation).^86^ Yet, in a war-torn economy where even mechanics frequently became unemployed, an increasing number of displaced people lived within the expanding reach of government aid that was sustained by internationalism in relief. They were not passive recipients of what was offered to them. I have shown that some, if not many, sought to acquire more from government aid schemes, or reshape the aid, by means such as exhibiting political awareness, producing patriotic narratives, mobilizing social elites to act for them and claiming refugee identity when necessary. In postwar Eastern Europe, the International Refugee Organization also witnessed the attempts by ethnic Germans to access its resettlement scheme, for example, by drawing on their multilingual background to claim another nationality.^87^ In the case of China, since the UNRRA aid was distributed through the state agency, the displaced population often chose to pressure the government through collective voices and actions. Having experienced waves of anti-foreign boycotts and protests that proved to be entangled with commercial interests, as well as official efforts at mass mobilization, they now exploited patriotism for their own purposes.^88^ Alert to the changing political situation and the stigma attached to refugees, they even included their own unpleasant presence into a pool of weapons with which to urge the revision of the government's aid provision.

After returning to his hometown in 1946, the famous artist Feng Zikai used a cartoon to portray a presumably common scene: a note saying ‘do not discuss political affairs’ was prominently placed in a teahouse.^89^ It expresses a sense of exhaustion among intellectuals after fearing for China's survival and future for over a decade. To a certain extent, having the option of not engaging in politics was a privilege, and elites would soon lose this privilege in the revolutionary Maoist era. In the two decades leading up to that era, displacement uprooted people from their place-based social networks. To them, creating and engaging with political identities was a survival tactic. Whereas native place, pre-war residence and affiliation with institutions formed connections that helped one endure war and displacement, war-induced political identities provided an uplifting and functional way to make sense of displacement, homelessness and unemployment.

These identities were often co-produced by the authorities and the displaced populations. As we have seen, newspapers and government regulations, as a media, facilitated the reinvention of righteousness from a traditional Confucian merit into a displacement identity, offering a foothold to turn government aid from a means of using human resources into a reward to loyalty. Compared to righteous people, the Nanyang mechanics was an exclusive identity, shared by a specific group of overseas Chinese and strengthened by both official arrangements and self-help practices. Indeed, it is common to see that government measures often helped form collective agency. To save administrative costs and cultivate democratic citizens, Nationalist officials divided the displaced men employed in state-based rehabilitation projects into groups and instructed them to elect leaders to serve as supervisors.^90^ People worked, lived, petitioned and protested together. They preferred to subsume individual experiences under collective narratives, sometimes opting for anonymity. This pattern was sharply different from what happened in postwar Europe, where individual narratives began to carry more weight because relief workers determined eligibility on a case-by-case basis.^91^ The international organizations that were created to seek solutions to displacement during and after the Second World War are often perceived as a homogenous entity that had a global operational scope, but they prompted different sorts of wisdom and agency to navigate aid provisions.

## Notes

1. ‘Nanmin yicheng yimin [Refugees Are Also Called Righteous People]’, 11 January 1939, *Shishi xinbao* (Chongqing), 3; though migrants sometimes emphasized their free will to follow the government's evacuation, rather than being forced to flee, in this article, I employ the term ‘displaced’ persons to refer to those whose relocation was ultimately because of the threat of war, and in this way to stress their vulnerability to war conditions in an unfamiliar place.

2. ‘Nanmin bushi yige chiru de mingci’, 13 January 1939, *Shishi xinbao*, 4*.*

3. R. MacKinnon, *Wuhan, 1938: War, Refugees, and the Making of Modern China* (Berkeley, CA 2008); H. Zurndorfer, ‘Wartime Refugee Relief in Chinese Cities and Women's Political Activism, 1937–1940’, in B. K. L. So and M. Zelin (eds) *New Narratives of Urban Space in Republican Chinese Cities: Emerging Social, Legal and Governance Order* (Leiden 2013), 65–91; M. S. Muscolino, ‘Refugees, Land Reclamation, Militarized Landscapes in Wartime China: Huanglongshan, Shaanxi, 1937–45’, *The Journal of Asian Studies*, 69, 2 (2010), 453–78.

4. R. K. Schoppa, *In a Sea of Bitterness: Refugees during the Sino-Japanese War* (Cambridge, MA 2011), 309–10.

5. L. Banko, K. Nowak and P. Gatrell, ‘What Is Refugee History, Now?’, *Journal of Global History* 17, 1 (2021), 1–19.

6. J. Reinisch, ‘Internationalism in Relief: The Birth (and Death) of UNRRA’, *Past and Present*, 210, supplement 6 (2011), 258–89; R. Mitter, ‘Imperialism, Transnationalism, and the Reconstruction of Post-War China: UNRRA in China, 1944–7’, *Past and Present*, 218, supplement 8 (2013), 51–69.

7. ‘Shanhou jiuji zongshu yewu zongbaogao [Final Report of CNRRA's Operations]’, April 1948, Q459-1-37, Shanghai Municipal Archives (Shanghai), 57.

8. H. van de Ven, *China at War: Triumph and Tragedy in the Emergency of the New China* (Cambridge 2018), 12.

9. R. Mitter, *China's War With Japan, 1937–1945: The Struggle for Survival* (London 2013); O. A. Westad, *Decisive Encounters: The Chinese Civil War, 1946–1950* (Stanford, CA 2003); G. Peterson, ‘To Be or Not to Be a Refugee: The International Politics of the Hong Kong Refugee Crisis, 1949–55’, *The Journal of Imperial and Commonwealth History*, 36, 2 (2008), 171–95; L. Madokoro, *Elusive Refuge: Chinese Migrants in the Cold War* (Cambridge, MA 2016).

10. Zurndorfer, ‘Wartime Refugee Relief in Chinese Cities and Women's Political Activism’, 68.

11. H. van de Ven, ‘Wartime Everydayness: Beyond the Battlefield in China's Second World War’, *Journal of Modern Chinese History*, 13, 1 (2019), 1–23.

12. Schoppa, *In a Sea of Bitterness*, 306; Van de Ven, ‘Wartime Everydayness’, 16.

13. G. H. Hodel and S. Hodel, *The China Letter: My Journey – Chief Regional Medical Officer Assigned to UNRRA-Hankow 1946* (Los Angeles, CA 2020), 24–5.

14. For portraits of refugees by Chinese elites of the time, see Y. Feng and Q. Situ, *Jiehou xing: yijiusiliu nian wusheng zaiqing jishi* [*In the Wake of Disaster: A Journey Across Five Provinces in 1946*] (Beijing, 2016).

15. For example, C. Henriot, ‘Shanghai and the Experience of War’, *European Journal of East Asian Studies*, 5, 2 (2006), 244.

16. On the concept of proxy agency, see A. Bandura, ‘Toward a Psychology of Human Agency: Pathways and Reflections’, *Perspectives on Psychological Science*, 13, 2 (2018), 130–6, 131.

17. C. Zou, ‘You yi er fei: qianxi kejia “yimin” shenfen de bianhua: jiyu Qingdai Taiwan he Guangdong xilu de bijiao yanjiu [From Righteousness to Banditry: A Comparative Study of the Changes of Hakka “Yimin” Identity in Taiwan Province and West Guangdong in Qing Dynasty]’, *Journal of Hebei Normal University (Philosophy and Social Sciences)* 45, 4 (2022), 37–44; J. Xiang, ‘Jingbiao yimin yu Mingdai de xiangcun zhili [Righteous People as Imperial Honors and Rural Governance in the Ming Dynasty]’, *Journal of Southwest University* (*Social Science Edition*), 46, 5 (2020), 164–75.

18. M. Ding, ‘Some Questions Concerning the Appraisal of the Boxer Movement’, *Chinese Studies in History*, 20, 3–4 (1987), 24–41, 26.

19. ‘Lun yimin yu luanmin zhi yi [On the Difference Between Yimin and Bandits]’, *Benguan lunshu*o, 52 (1900), 3327–32.

20. Z. Chen, *Jiang Tingfu de zhishi yu pingsheng* [*Jiang Tingfu's Aspirations and Life Story*] (Taipei 1967), 15–17.

21. J. Oldstone-Moore, ‘Confucianism in Republican China, 1911–1949’, in J. Oldstone-Moore (ed.) *The Oxford Handbook of Confucianism* (New York, NY 2023), 204–16, 210–11.

22. C. K. Chiang, *Xinshenghuo yundong gangyao* [*Outline of the New Life Movement*] (Taipei 1945), 4–6.

23. ‘Xinshenghuo yu minzu fuxing [The New Life and National Reconstruction]’, 25 February 1936, *Shishi xinbao* (Shanghai), 2.

24. ‘Jiuji dongbei nanmin [Relief to Refugees from the Northeast]’, 9 November 1932, *Shishi xinbao*, 3; ‘kegekeqi de yimin de yi feng xin [A Heroic and Moving Letter Written by Righteous People]’, 7 April 1933, *Xinwen bao* (Shanghai), 10; ‘Shisi kangdi de yimin [Righteous People Resisting the Enemy and Swearing to Death]’, 21 June 1937, *Ertong chenbao* (Shanghai), 3.

25. R. Mitter, *The Manchuria Myth: Nationalism, Resistance, and Collaboration in Modern China* (Berkeley, CA 2000), 175.

26. ‘Xu Shiying jiangshu jiuji fangan [Xu Shiying Talks about Relief Schemes]’, 22 May 1938, *Shenbao* (Hankou), 2; MacKinnon, *Wuhan, 1938*, 47–9.

27. ‘Yao shi nanmin cheng yimin [Refugees Shall be Transformed into Righteous People]’, 27 September 1938, *Xinwen bao* (Shanghai), 5.

28. L. Watt, *When Empires Come Home: Repatriation and Reintegration in Postwar Japan* (Cambridge, MA 2009), 16–17; on public concerns over refugees’ hygienic conditions, see ‘Nanmin weisheng [Refugee Hygiene]’, 11 June 1938, *Daobao* (Shanghai), 5.

29. ‘Sichuan ren xinmu zhong de xiajiangren [Downriver People in the Eyes of Sichuan People]’, 7 April 1940, *Damei zhoubao* (Shanghai), 10; for more cases on downriver people, see Danke Li, *Echoes of Chongqing: Women in Wartime China* (Urbana and Chicago, IL 2010).

30. For example, see, ‘Nanmin kangdi xuanshi [Refugees Swear to Resist the Enemy]’, 25 April 1939, *Shishi xinbao*, 3.

31. Z. Wan and J. He, ‘Liangnan de kunjing: Kangzhan shiqi de nanmin huiliu wenti (1938–1940) [The Dilemma of Refugee Return During the War of Resistance Against Japanese Aggression]’, *Journal of Japanese Invasion of China and Nanjing Massacre*, 2 (2023), 41–52.

32. ‘Nanmin bushi yige chiru de mingci’, 13 January 1939, *Shishi xinbao*, 4*.*

33. ‘Sichuansheng zhenji weiyuanhui xunling [Order Issued by the Relief Committee in Sichuan Province]’, May 1940, 00810004000830000010000, Chongqing Municipal Archives (Chongqing).

34. ‘Yimin gongzuo liangyue [Two-Month Refugee Relief Work]’, *Guangxi funü*, 1, 11 (1940), 24–5; ‘Shenru nanminqun [Reaching Refugees]’, 18 December 1938, *Dagong bao* (Chongqing), 3.

35. ‘Yimin tanfan zuzhi huzhuhui [Righteous People Who Make a Living as Hawkers Organised a Self-Help Association]’, *Dagong bao* (Guilin), 7 September 1942, 3; also note that a *zhanqu yimin huzhuhui* [Yimin's Self-Help Association in the War Zone] was established in Chongqing in March 1939, see Chongqing Municipal Archives and Chongqing Normal University (eds) *Zhongguo zhanshi shoudu dangan wenxian: zhanshi shehui* [Archival Sources About China's Wartime Capital: Wartime Society] (Chongqing 2014), 95.

36. CNRRA Hubei Office to Yichang Refugee Station, 12 March 1946, LS030-001-0254-0008, Hubei Provincial Archives (Wuhan).

37. See *Helping the People to Help Themselves: The Story of the United Nations Relief and Rehabilitation Administration* (London 1944), 2.

38. ‘Shanhou jiuji zongshu yewu zongbaogao’, 57.

39. ‘Bushi maimai nanmin zheng [The Transaction of Refugee Certificates Should not be Allowed]’, 3 November 1945, *Zhongyang ribao* (Chongqing), 5; note that the Nationalist government had run an ID-based refugee management system in wartime, see Mitter, ‘Imperialism, Transnationalism, and the Reconstruction of Post-War China’, 56.

40. ‘Wuhan linshi jiuji weiyuanhui gongzuo zongbaogao [Final Report of the Wuhan Temporary Relief Committee]’, 20 July 1946, LS006-002-0634-0013, Hubei Provincial Archives.

41. ‘Shehui bu ge zhishu shehui fuwu jigou banli yimin fanxiang fuwu gongzuo banfa [Regulations on Handling the Return of Righteous People by Social Service Institutions Affiliated With the Ministry of Social Affairs]’, 28 December 1946, *Zhejiang jingji yuekan* 2, 2 (1947), 91.

42. ‘Nanmin de jia: Wan shou gong [Refugees’ Home: The Wan Shou Temple]’, 12 July 1946, *Xinhua ribao* (Chongqing), 3.

43. ‘Yimin de shensu [Righteous People’ Appeal]’, *Shenbao*, 15 May 1947, 7.

44. Ibid.

45. B. Kushner, ‘Ghosts of the Japanese Imperial Army: The “White Group” (baituan) and Early Post-War Sino–Japanese Relations’, *Past and Present*, 218, supplement 8 (2013), 117–50.

46. ‘Shanhou jiuji zongshu yewu zongbaogao’, 66; Woodbridge, *UNRRA*, 411.

47. ‘Yimin de shensu’, 7.

48. Mitter, ‘Imperialism, Transnationalism, and the Reconstruction of Post-War China’, 59; T. Ma, ‘A Chinese Beveridge Plan: The Discourse of Social Security and the Postwar Reconstruction of China’, *European Journal of East Asian Studies,* 11, 2 (2012), 329–49; R. Mitter, ‘State-building after Disaster: Jiang Tingfu and the Reconstruction of Post-World War II China, 1943–1949’, *Comparative Studies in Society and History*, 61, 1 (2019), 176–206.

49. MacKinnon, *Wuhan, 1938*, 57–60; Henriot, ‘Shanghai and the Experience of War’, 219.

50. F. Ferlanti, ‘Women's Activism and Mobilization in Wartime China: Cadre Training, National Economic Production, and Workers’ Literacy (1937–1945)’, *Journal of Chinese History*, 9 (2025), 62–83, 67, 74–9; ‘Yimin gongzuo liangyue’, 24.

51. ‘Yimin de shensu’, 7.

52. ‘Wangshi huiyi zhongzhong [Recollections of the Past]’, *Shenbao*, 10 July 1946, 8.

53. ‘Yimin de shensu’, 7.

54. Representatives of the Refugees in the Kiangsu Building, Hankow, to UNRRA Hankow Regional Office, 10 April 1946, S-1148-0000-0114, United Nations Archives and Records Management Section.

55. D. M. Yang, ‘The Displacement and Relief for Chiang Kai-shek's “Righteous Compatriots” in the Global Cold War’, *The Historical Journal*, 68 (2025), 667–88.

56. E. Koh, *Diaspora at War: The Chinese of Singapore Between Empire and Nation, 1937–1945* (Leiden 2013), 68–76; M. Zhuang, ‘Chen Jiageng yu Huaqiao jigong [Tan Kah-Kee and Overseas Chinese Mechanics]’, in Q. Qin and J. Tang (eds) *Nanqiao jigong huiguo kangrishi* [*History of Overseas Chinese Mechanics Returning to China to Fight Against Japan*] (Kunming 1989), 1–10.

57. Mark R. Frost, ‘Humanitarianism and the Overseas Aid Craze in Britain's Colonial Straits Settlements, 1870–1920’, *Past & Present*, 236 (2017), 169–205, 179–89; C. F. Yong, *Tan Kah-kee: The Making of an Overseas Chinese Legend* (Singapore 2013), 178–228.

58. ‘More mechanics for China’, 20 March 1939, *The Singapore Free Press and Mercantile Advertiser* (Singapore), 3.

59. See ‘Nanqiao jigong [South Seas Overseas Chinese Mechanics]’, 2014, China Central Television. Available at https://jishi.cntv.cn/special/nqjg/index.shtml; regarding Taiwan, in 2015, descendants of the overseas Chinese mechanics were invited to Taiwan to a government-run commemoration event for China in the Second World War.

60. S. Lin, *Fenghuo chizi xin: Dianmian gonglu shang de nanqiao jigong* [*Patriotism in Wartime: Nanayang Overseas Chinese on the Burma Road*] (Beijing 2015), 1.

61. For example, see Qin and Tang (eds), *Nanqiao jigong huiguo kangrishi*.

62. Koh, *Diaspora at War*, 77.

63. J. Li, ‘Kangzhan shiqi Xinan yunshu de fazhan yu kunjing: yi Dianmian gonglu wei zhongxin de tantao [Wartime Transport Administration in Southwest China and the Burma Road (1938–1942)]’, *Bulletin of Academia Historica*, 33 (2012), 57–88, 61–70.

64. Nearly 1.5 million people from today's Southeast Asia and Hong Kong were registered with the Nationalist government in 1943, see M. Oyen, ‘The Right of Return: Chinese Displaced Persons and the International Refugee Organization, 1947–56’, *Modern Asian Studies*, 49, 2 (2015), 546–71, 552.

65. Z. Zhang, ‘Kang wokou qiaogong sa rexue, bao Zhonghua chizi biao danxin [Overseas Chinese Mechanics Struggled to Resist Japan for Patriotism]’, 10 June 1985, in Qin and Tang (eds), *Nanqiao jigong huiguo kangrishi*, 200–8, 203; CNRRA Bureau of Relief to UNRRA Shanghai Office, 9 August 1946, see P. Hsie (ed.), *Zhanhou qianfan huaqiao shiliao huibian* [*A Documentary Collection of Post-Second World War Repatriation of Overseas Chinese*] (Taipei 2005), vol. 3, 345–7.

66. Y. Xia, *Nanyang huaqiao jigong yanjiu* [*A Study of the Nanyang Volunteer Drivers and Mechanics*] (Beijing 2016), 165.

67. See *Huaqiao jigong tongxunkan*, 40 (1940), 1–2; ‘Xiangyan chou bude [One Shall Not Smoke Cigarettes]’, *Huaqiao jigong tongxunkan*, 11 (1940), 1.

68. Xia, *Nanyang huaqiao jigong yanjiu*, 162–87.

69. On a similar case of collective identity in Second World War Asia, see T. M. Chen, ‘Chinese Residents of Burma as Refugees, Evacuees, and Returnees: The Shared Racial Logic of Territorialization in the Regulation of Wartime Migration’, *Modern Asian Studies*, 49, 2 (2015), 469–92, 470–3.

70. ‘Diansheng huaqiao huzhuhui baogaoshu [Report by the Overseas Chinese Self-Help Association in Yunnan Province]’, *Zhongyang ribao* (Chongqing), 4 December 1944, 3.

71. Ibid.

72. ‘Zhang Mingli baogao huaqiao jigong shikuang’, 30 May 1946, 2; some of the Nanyang mechanics married local women and had children, see P. Hsie (ed.), *Zhanhou qianfan huaqiao shiliao huibian* [*A Documentary Collection of Post-Second World War Repatriation of Overseas Chinese*] (Taipei 2005), vol. 3, 409–16.

73. Chen Shuren to the Ministry of Foreign Affairs, 2 November 1945, in Hsie (ed.), *Zhanhou qianfan huaqiao shiliao huibian*, vol. 3, 283–5.

74. Chen Shuren to the Ministry of Foreign Affairs, 2 November 1945, in Hsie (ed.), *Zhanhou qianfan huaqiao shiliao huibian*, vol. 3, 284–5.

75. Oyen, ‘The Right of Return’, 554–5.

76. Guomin canzhenghui disijie dierci dahui ti’an yuanwen [Proposals Submitted in the Fourth Session, Second Meeting of the National Political Consultative Conference] (Chongqing: Secretariat of the National Political Consultative Conference, 1946), 107–8; see also ‘Nanyang qiaogong qingyuan fuyuan [Nanyang Mechanics Petitioned for Repatriation]’, 29 March 1946, *Shenbao*, 1.

77. ‘Huiguo jigong liulishisuo, Quanma qiaobao biao fenkai [All Overseas Chinese Across British Malaya are Outraged at Returned Mechanics’ Homelessness]’, *Qiaosheng bao*, 2 July 1947, 2; Xia Yuqing's research also presents a detailed account of Tan Kah-kee's negotiations with China's consul in Singapore, Xia, *Nanyang huaqiao jigong yanjiu*, 303–9.

78. ‘Kangzhan yougong zhi Nanqiao jigong bei shou Jiangjia zhengfu nüedai [The Nanyang Mechanics Who Have Contributed to China's War Effort are now Abused by Chiang Kai-Shek's Government]’, 21 July 1946, *Jin ji cha ribao* (Yan’an), 3.

79. ‘Dian Huaqiao huzhuhui kai jigong fuyuan hui [The Overseas Chinese Self-Help Association in Yunnan Province Held a Meeting on the Repatriation of Mechanics]’, *Xinsheng bao*, 20 September 1946, 1.

80. CNRRA Bureau of Relief to UNRRA Shanghai Office, 9 August 1946, in Hsie (ed.), *Zhanhou qianfan huaqiao shiliao huibian*, vol. 3, 345–7; also see Jayne Persian, ‘Displaced Persons and the Politics of International Categorisation(s)’, *Australian Journal of Politics & History*, 58, 4 (2012), 481–96, 482.

81. Ministry of Foreign Affairs to Overseas Chinese Affairs Commission, 16 November 1946, in Hsie (ed.), *Zhanhou qianfan huaqiao shiliao huibian*, vol. 3, 406.

82. Refugee Representatives of the CNRRA Second Refugee Camp to the Shanghai City Council, 26 October 1947, Q109-1-1702, Shanghai Municipal Archives.

83. On the struggle of refugees fleeing the CCP rule in northern Jiangsu province into Shanghai, see J. Y. Chen, ‘Will the Real Refugees Please Stand Up? War, Revolution, and the Politics of the Subei Refugee Crisis, 1940–1948’, *Twentieth-Century China*, 38, 2 (2013), 99–118.

84. For example, see the case in Zhejiang province, ‘Houfang fanzhe yimin, luxu zisong yuanji [Righteous People From the Rear Returned to Zhejiang Province and Were Gradually Sent to Their Native Places]’, *Shenbao*, 22 June 1946, 2.

85. ‘Report on the Wuhan Refugee Control Committee’, 15 October 1946, S-1148-0000-0050, United Nations Archives and Records Management Section.

86. For example, see K. von Lingen, ‘Citizenship, Expropriation, and Redress: “Migrating Objects” and the Case of Holocaust Victims from Austria’, *The Historical Journal*, 68 (2025), 518–39, 527; K. Nowak, *Kingdom of Barracks: Polish Displaced Persons in Allied-Occupied Germany and Austria* (London 2023), 161–97.

87. J. Panagiotidis, ‘“Not the Concern of the Organization?” The IRO and the Overseas Resettlement of Ethnic Germans from Eastern Europe after World War II’, *Historical Social Research*, 45, 4 (2020), 173–202.

88. See G. Wang, *In Search for Justice: The 1905–1906 Chinese Anti-American Boycott* (Cambridge, MA 2001); K. Gerth, *China Made: Consumer Culture and the Creation of the Nation* (Cambridge, MA and London 2003); F. Ferlanti, ‘The New Life Movement at War: Wartime Mobilisation and State Control in Chongqing and Chengdu, 1938–1942’, *European Journal of East Asian Studies*, 11, 2 (2012), 187–212.

89. C. Feng and Y. Feng (eds), *Feng Zikai manhua quanji* [*The Complete Collection of Feng Zikai*] (Beijing 2001), vol. 4, 68; on Feng Zikai's displacement experience, see Schoppa, *In a Sea of Bitterness*, 59–86.

90. Shanghai Winter Relief Commission to the Thirteenth Work-Relief Camp, 1 February 1948, Q106-1-15-58, Shanghai Municipal Archives.

91. G. D. Cohen, ‘Between Relief and Politics: Refugees Humanitarianism in Occupied Germany, 1945–1946’, *Journal of Contemporary History*, 43, 3 (2008), 437–449, 446–7.

